# Health Literacy and Capecitabine Adherence in a Remote Monitoring Pilot Trial for Breast Cancer: Post Hoc Exploratory Analysis

**DOI:** 10.2196/101210

**Published:** 2026-09-14

**Authors:** Samuel Hernandez, Jane Meisel, Gelareh Sadigh, Ilana Graetz

**Affiliations:** 1 Medical College of Georgia Augusta, GA United States; 2 Winship Cancer Institute Atlanta, GA United States; 3 School of Medicine Department of Radiological Sciences University of California, Irvine Irvine, CA United States; 4 Health Policy and Management Rollins School of Public Health Emory University Atlanta, GA United States

**Keywords:** health literacy, remote patient monitoring, breast cancer, adherence, mobile health, mHealth, capecitabine

## Abstract

**Background:**

Oral anticancer therapy enables convenient, home-based cancer care but can introduce adherence challenges, particularly with complex dosing schedules. Capecitabine is commonly used in breast cancer, often as adjuvant therapy or in advanced disease, and typically requires twice-daily dosing on cyclical schedules, increasing the risk of missed or incorrect doses. Low health literacy may exacerbate these difficulties, and emerging remote monitoring tools may help close this gap.

**Objective:**

In this post hoc exploratory analysis, we evaluated whether health literacy (1) was associated with capecitabine adherence and (2) modified a remote monitoring intervention’s effectiveness.

**Methods:**

We conducted post hoc analyses of a 2-arm pilot trial that randomized women with breast cancer treated with capecitabine to enhanced usual care (EUC) or remote patient monitoring (RPM). Adherence was captured with a smart pill bottle, Nomi by SMRxT, that recorded dose timing and quantity. Participants in the RPM group received messages for missed or incorrect doses and weekly symptom assessments. Incorrect or missed doses and severe symptoms triggered alerts to the oncologist. Health literacy was assessed at enrollment. To evaluate moderation, we used linear regression with an interaction term (health literacy × intervention arm) predicting adherence (proportion of days). Marginal effects quantified differences in adherence by study arm and health literacy.

**Results:**

Among 28 participants (EUC, n=15 and RPM, n=13), 9 (32.1%) had lower health literacy, 16 (57.1%) identified as Black, 10 (35.7%) identified as White, and 15 (53.6%) had income below 200% of the federal poverty level. In the regression model, the health literacy × randomized group interaction did not reach statistical significance (−16.3 percentage points, 95% CI −35.5 to 2.9; *P*=.09). Predicted adherence among lower health literacy participants was 87.5% in the RPM group and 65.5% in the EUC group (difference: +22.1 percentage points, 95% CI 6.2-37.9; *P*=.008). Among participants with higher health literacy, adherence was 89.9% in the RPM group and 84.1% in the EUC group (difference: +5.7 percentage points, 95% CI −5.2 to 16.7; *P*=.29). Within the EUC group, predicted adherence was 18.6 percentage points lower among those with lower versus higher health literacy (95% CI −32.4 to −4.9; *P*=.01); within the RPM group, this difference was 2.3 percentage points lower among those with lower versus higher health literacy (95% CI −15.8 to 11.1; *P*=.73).

**Conclusions:**

In this post hoc exploratory analysis, the estimated difference in capecitabine adherence between the RPM and EUC groups was larger among participants with lower health literacy. Although the formal interaction test was not statistically significant, the magnitude and direction of the observed difference support further investigation of RPM as a potential approach to improve adherence among patients facing health literacy–related adherence barriers. Larger, prospectively powered studies are needed to confirm these findings and evaluate downstream clinical outcomes.

## Introduction

Oral anticancer regimens have noninferior outcomes among patients with cancer while improving quality of life and overall tolerability [1]. It allows patients to manage their treatments at home, reducing the need for frequent clinic visits, such as those needed for intravenous administration [2]. At the same time, shifting administration from clinic to home introduces new vulnerabilities to treatment efficacy and safety, particularly for medications with complex dosing schedules or frequent dose modifications.

Capecitabine is commonly used across multiple breast cancer settings, with specific indications and lines of therapy varying by tumor subtype and stage [3,4]. Dosing schedules are complex, requiring multiple tablets twice daily on cyclical schedules, typically given either in 21-day cycles (14 days on treatment and 7 days off) or in 14-day cycles (7 days on treatment and 7 days off) [5,6]. In the adjuvant setting, patients are treated for 6 to 8 cycles, whereas in the metastatic setting, patients remain on treatment until disease progression or unacceptable toxicity. Dose modifications are commonly due to drug toxicities, particularly hand-foot syndrome and diarrhea. Medication adherence is the extent to which a patient’s medication-taking behavior is consistent with their health care provider’s advice [7]. Capecitabine requires an adherence rate of >90% to optimize efficacy [8], but reported rates range from 78.1% to 97.9% [9-11]. Common reasons for nonadherence include adverse symptoms, forgetting to take doses, and misunderstanding dose instructions [8,12,13].

Oral chemotherapy shifts treatment responsibility to patients, who must manage complex dosing schedules and side effects at home with limited clinical oversight. Health literacy, defined as the combination of personal competencies and situational resources needed for people to access, understand, appraise, and use information and services to make decisions about health, directly influences self-management capacity [14]. It represents the interplay of how health care providers communicate information and how patients can access, understand, and use that information to make informed decisions [15,16]. Lower health literacy can impair a patient’s understanding of their treatment, including the benefit of high adherence [8]. This may result in suboptimal adherence and ultimately worse and potentially preventable outcomes [8,17]. As oncological care shifts toward oral chemotherapy regimens, there is an increased need for health systems to address barriers such as low health literacy. Research is needed to understand the specific barriers and facilitators impacting treatment adherence, with tiered, individualized remote monitoring interventions tailored for patient support.

We conducted an exploratory post hoc analysis of a randomized pilot trial that demonstrated improvements in adherence, mental health quality of life, and symptom burden using a novel remote patient monitoring (RPM) mobile health (mHealth) intervention with a smart pill bottle and supportive text messaging among women with breast cancer taking capecitabine [18]. Building on prior app-based remote monitoring trials for adjuvant endocrine therapy [19], this post hoc analysis evaluates the role of health literacy in adherence and intervention response among patients taking capecitabine, an oral chemotherapy with a complex regimen, using a simpler, app-free platform that combines a smart pill bottle with text messages.

## Methods

### Study Design and Participants

The nonblinded randomized controlled pilot trial (NCT05086731) evaluated the feasibility and effectiveness of an RPM mHealth intervention at a multisite cancer center using a smart pill bottle, Nomi (developed by our research partner SMRxT). Women with a breast cancer diagnosis and a capecitabine (brand name: Xeloda) prescription receiving care at a National Cancer Institute (NCI)–designated comprehensive cancer center or at a large public hospital between December 2021 and May 2023 were eligible for this trial. Participants who provide consent were randomized into one of two arms: (1) enhanced usual care (EUC)—receiving usual care, including routine follow-ups with their oncology team, or (2) RPM—receiving text reminders if they missed their scheduled dose by more than 2 hours or took more or fewer pills than prescribed, reporting symptoms weekly, and receiving an adherence report at the end of each cycle. Reports of severe symptoms or greater than or equal to 10% missed or incorrect doses within a 7-day period triggered an alert to the patient’s medical oncology team. Participants in both the EUC and RPM groups were asked to use the smart pill bottles for up to 3 capecitabine pill cycles and complete a survey at enrollment and study completion.

### Ethical Considerations

The study was approved by the Emory University Institutional Review Board (STUDY00002985), and the study procedures have been described previously [18]. Informed consent was obtained from all participants by the study coordinator, and participants could opt out of the study at any time. Privacy and confidentiality were maintained by collecting only patient initials, phone number, and medication information on the secure smart pill bottle platform. Study data were stored in a secure, locked electronic file and accessed only by the institutional review board–approved study team. Data used for analyses were deidentified. Participants received US $20 and US $40 gift cards after completing the enrollment and follow-up surveys, respectively.

### Baseline Measures and Study Outcomes

An enrollment survey captured participants’ sociodemographic characteristics, including race or ethnicity, income level (≥200% vs <200% of the federal poverty level [FPL]), stage (early vs metastatic), and education (less than a bachelor’s degree vs more than a bachelor’s degree).

Adherence was captured with the smart pill bottle and calculated as the proportion of days with the correct amount of medication taken in the day, regardless of time, divided by the number of days monitored, excluding health care provider–initiated treatment pauses. Treatment adherence was measured for all participants who received the intervention (ie, used the smart pill bottle system). The bottle recorded changes in bottle weight as a proxy for medication use; it did not independently distinguish patient-initiated nonadherence from health care provider–directed dose reductions, schedule changes, or temporary treatment holds. To reduce misclassification, the study coordinator reconciled prescription changes during follow-up with the oncology team and participant, and health care provider–directed dose changes or pauses were excluded from nonadherence calculations.

Health literacy was measured at enrollment using a validated single-item question: “How confident are you filling out forms by yourself?” [20]. Responses were measured using a 5-point Likert scale and categorized as higher (always) versus lower health literacy (never, rarely, sometimes, and often). This single-item screener has been validated across diverse clinical populations and correlates strongly with comprehensive health literacy instruments [20,21].

Health beliefs were assessed using 7 statements addressing potential barriers to and facilitators of adherence, rated on a 5-point Likert scale (1=strongly agree to 5=strongly disagree). Statements were adapted from a validated survey assessing facilitators of and barriers to mammography screening [22]. We also created indicator variables for reporting any adherence barrier or facilitator. Barriers to adherence were assessed using the following statements: “I find it inconvenient to get my Xeloda (capecitabine) prescription filled on a regular basis.”; “Taking my Xeloda (capecitabine) medication is difficult because it costs too much money.”; “Taking my Xeloda (capecitabine) medication is difficult because it causes uncomfortable side effects.”; “I have difficulty remembering to take Xeloda (capecitabine) medication.” Facilitators of adherence included the following statements: “I understand why I was prescribed Xeloda (capecitabine) and how it works.”; “My doctor adequately explained how I should take my Xeloda (capecitabine) medication and for how long.”; “Taking my Xeloda (capecitabine) medication exactly as prescribed will decrease the chance of my breast cancer coming back.”

### Statistical Analysis

Categorical participant characteristics and reported adherence barriers and facilitators were compared by health literacy using the Fisher exact test because of small cell sizes. The mean number of capecitabine pills prescribed per day was compared between study arms and by health literacy using 2-sample, 2-sided *t* tests assuming equal variances. To evaluate whether health literacy modified intervention effectiveness, we fit a linear regression model with adherence as the dependent variable and included health literacy, intervention arm, and their interaction as predictors; no additional covariates were included given the small pilot sample size. We assessed model assumptions by examining residual plots and the distribution of fitted values and considered the bounded nature of adherence in selecting a linear model for this exploratory analysis. Marginal effects were then used to estimate adjusted differences across health literacy levels and intervention groups. The analyses used Stata (version 18; StataCorp).

## Results

Among the 32 participants who provided consent, 28 received the intervention (EUC, n=15 and RPM, n=13) and had adherence data captured by smart pill bottles (Table 1). Most participants identified as Black (16/28, 57.1%), 35.7% (n=10) identified as White, and 53.6% (n=15) had income below 200% of the FPL. A total of 9 (32.1%) participants had lower health literacy, including 4 in the EUC group and 5 in the RPM group (*P*=.69). All participants with lower health literacy were aged >50 years compared to 63.2% (12/19) of those with higher health literacy (*P*=.06). Health literacy did not differ substantially by race or ethnicity, education, income, cancer stage, or study arm.

More than half (7/13, 53.9%) of the participants in the RPM group and 33.3% (5/15) of the participants in the EUC group had stage IV disease (*P*=.45). The mean number of pills prescribed per day was 5.7 (SD 1.4; range 2-8) and did not differ significantly by study arm (*P*=.39) or health literacy (*P*=.89). There were no statistically significant differences in baseline participant characteristics by study arm.

While there were no statistically significant differences in self-reported adherence barriers or facilitators by health literacy status, some trends are notable (Figure 1). Participants with lower health literacy more often reported that filling the medication was inconvenient (3/9, 33.3% vs 2/18, 11.1%; *P*=.30), whereas those with higher health literacy more often reported forgetfulness (2/19, 10.5% vs 0/9, 0%; *P*=.99). Cost barriers were similar (2/19, 10.5% higher health literacy vs 1/9, 11.1% lower health literacy; *P*=.99). Higher health literacy participants more often reported at least 1 adherence facilitator (19/19, 100% vs 7/9, 77.8%; *P*=.09), with consistently higher frequencies for individual facilitators.

In the regression model examining capecitabine adherence (Figure 2), including randomized group, health literacy category, and their interaction, the interaction term did not reach statistical significance (−16.3 percentage points, 95% CI −35.5 to 2.9; *P*=.09).

Model-based predicted adherence among participants with lower health literacy was 87.5% in the RPM group and 65.5% in the EUC group, corresponding to an estimated RPM-EUC difference of 22.1 percentage points (95% CI 6.2-37.9; *P*=.008). Among participants with higher health literacy, predicted adherence was 89.9% in the RPM group and 84.1% in the EUC group, corresponding to an estimated RPM-EUC difference of 5.7 percentage points (95% CI –5.2 to 16.7; *P*=.29).

Within the EUC group, predicted adherence was lower among participants with lower versus higher health literacy (65.5% vs 84.1%; difference: −18.6 percentage points, 95% CI −32.4 to −4.9; *P*=.01). Within the RPM group, predicted adherence was similar among participants with lower and higher health literacy levels (87.5% vs 89.9%; difference: −2.3 percentage points, 95% CI −15.8-11.1; *P*=.73).

**Table 1:** Baseline characteristics of women with breast cancer who were prescribed capecitabine and enrolled in a remote monitoring pilot randomized trial, stratified by health literacy (N=28).^a^

		Overall, n (%)	Higher health literacy (n=19), n (%)	Lower health literacy (n=9), n (%)	*P* value	
Age (years)						.06
	≤50	7 (25)	7 (36.8)	0 (0)		
	>50	21 (75)	12 (63.2)	9 (100)		
Race						.19
	Asian	1 (3.6)	1 (5.3)	0 (0)		
	Black	16 (57.1)	11 (57.9)	5 (55.6)		
	White	10 (35.7)	6 (31.6)	4 (44.4)		
	Missing	1 (3.6)	1 (5.3)	0 (0)		
Education						.65
	Less than a bachelor’s degree	7 (25)	4 (21.1)	3 (33.3)		
	More than a bachelor’s degree	21 (75)	15 (79)	6 (66.7)		
Income, % FPL^b^						.44
	<200	15 (53.6)	9 (47.4)	6 (66.7)		
	≥200	13 (46.4)	10 (52.6)	3 (33.3)		
Stage						.43
	Early	16 (57.1)	12 (63.2)	4 (44.4)		
	Metastatic	12 (42.9)	7 (36.8)	5 (55.6)		
Randomization arm						.69
	EUC^c^	15 (53.6)	11 (57.9)	4 (44.4)		
	RPM^d^	13 (46.4)	8 (42.1)	5 (55.6)		

^a^Health literacy was assessed using the item, “How confident are you filling out medical forms by yourself?” Lower health literacy was defined as responses of never, rarely, sometimes, or often, whereas higher health literacy was defined as a response of always. FPL was calculated based on the US Department of Health and Human Services FPL guidelines. *P* values were calculated using 2-sided Fisher exact tests.

^b^FPL: federal poverty level.

^c^EUC: enhanced usual care.

^d^RPM: remote patient monitoring.

**Figure 1:**
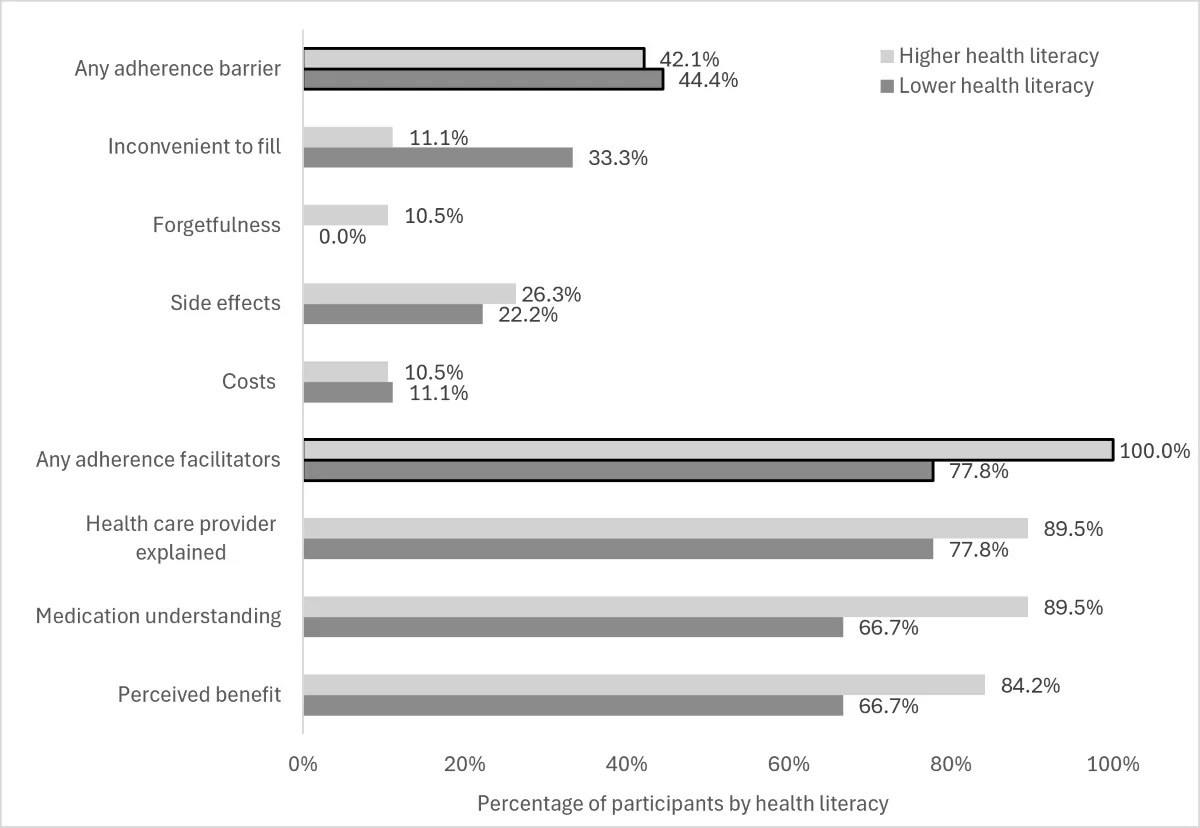
Reported medication adherence barriers and facilitators by health literacy among women with breast cancer prescribed capecitabine and enrolled in a remote monitoring pilot randomized trial.

**Figure 2:**
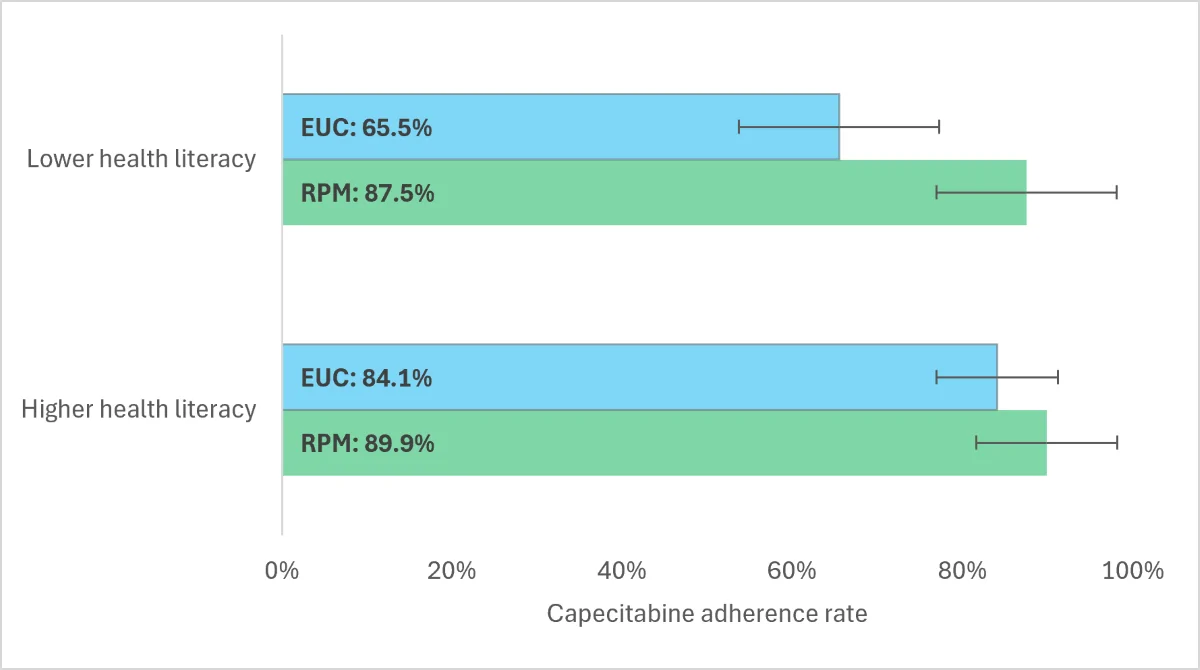
Model-based predicted capecitabine adherence by health literacy and study arm among women with breast cancer prescribed capecitabine and enrolled in a remote monitoring pilot randomized trial. A linear regression model with health literacy, study arm, and an interaction term (health literacy × study arm) was used to estimate predicted adherence by health literacy and study arm. Error bars represent 95% CIs. The interaction term estimate did not reach statistical significance (−16.3 percentage points; 95% CI −35.5 to 2.9; *P*=.09). *P*=.008 for the remote patient monitoring (RPM) vs enhanced usual care (EUC) comparison within the lower health literacy subgroup and *P*=.29 for the corresponding comparison among participants with higher health literacy.

## Discussion

### Principal Findings

In this post hoc exploratory analysis of a pilot trial among women with breast cancer who were prescribed capecitabine, lower health literacy was associated with poorer adherence in the EUC group but not in the RPM group. RPM was associated with significantly higher adherence among participants with lower health literacy, whereas adherence did not differ meaningfully by the intervention arm among those with higher health literacy. Although the formal interaction test did not reach statistical significance, the magnitude and direction of the observed difference support further investigation of RPM as a potential approach to improve adherence among patients facing health literacy–related barriers.

### Comparison With Prior Work

Our findings are consistent with those of a prior study showing that an app-based remote monitoring intervention with tailored text messages improved adherence to adjuvant endocrine therapy among patients with breast cancer and lower health literacy but had no effect among those with higher health literacy [19]. This suggests that remote monitoring interventions may be particularly effective for supporting treatment adherence among individuals with lower health literacy. Although our study was smaller, it makes several distinct contributions: it is one of the first to evaluate a remote adherence monitoring intervention specifically for capecitabine, an oral chemotherapy with a more complex dosing schedule and distinct adherence challenges than adjuvant endocrine therapy, and it includes a substantially more diverse sample, with more than half of the participants identifying as Black and a higher proportion having relatively lower health literacy. In addition, the intervention was designed to be more accessible, using a simple smart pill bottle–based monitoring platform and short text messages rather than a smartphone-dependent web-based app, which may broaden its applicability to populations with variable digital access and literacy.

Participants with lower health literacy were found to have lower medication adherence when compared to participants with higher health literacy in the EUC arm, aligning with similar findings among low health literacy participants from prior analyses [19,23]. Previous studies have identified health literacy as an important factor of cancer self-management behaviors and survival outcomes, with individuals who have lower health literacy experiencing disproportionate challenges [15,24]. Low health literacy has been associated with a poorer understanding of health-related information and factors affecting health, which may adversely affect long-term outcomes and quality of life [15,16,24]. Inadequate health literacy has been associated with greater information needs and a lower likelihood of seeking answers to health-related questions [25]. In this study, participants with lower health literacy tended to report fewer adherence facilitators, potentially reflecting gaps in understanding dosing schedules and treatment benefit. Our findings provide preliminary evidence that treatment-related factors, including an understanding of treatment schedules and perceived treatment benefits, may represent important modifiable barriers to adherence among adults with lower health literacy and could be targeted through supportive interventions.

Evidence indicates that people with higher health literacy levels can play a more active role in their care and stay more informed about their condition and health status [24,26]. Participants with higher health literacy more frequently reported adherence facilitators, particularly a greater understanding of treatment, perceived treatment benefits, and health care provider explanations, highlighting potential differences from participants with lower health literacy. Although participants in the EUC group with higher health literacy had relatively higher adherence rates than those with lower health literacy (84.1% vs 65.5%), adherence remained suboptimal, indicating room for improvement [8]. This suggests that even among patients with adequate health literacy, additional adherence support, such as reminders for forgetfulness and symptom management for side effects, may be needed to reach optimal adherence. While participants with higher health literacy reported more adherence facilitators, they also reported certain barriers more often than those with lower health literacy, such as forgetfulness related to medication taking and experiencing burdensome side effects. These findings suggest the possible need for patient-centered interventions tailored to address the dominant barriers experienced by different patient subgroups.

### Implications for Clinical Practice

RPM and related digital monitoring strategies may offer a practical approach to supporting adherence to oral anticancer therapy, particularly when adherence barriers are identified early and monitoring is linked to a timely clinical response [27-32]. Recent evidence from a meta-analysis suggests that digital oncology interventions can improve symptom management and may support adherence, but evidence for adherence itself remains heterogeneous, and implementation costs, staffing requirements, and reimbursement pathways remain limited [31]*.* RPM programs require substantial upfront investment, including the use of secure, Health Insurance Portability and Accountability Act (HIPAA)–compliant platforms; development of processes for patient enrollment, education, and technical support; integration of patient- and device-generated data into the electronic health record (EHR); and clearly specified escalation algorithms for missed doses, worsening symptoms, or other clinically actionable reports. The adoption of RPM accelerated following the introduction of the Centers for Medicare and Medicaid Services reimbursement codes in 2018 to 2019, with further expansion during the COVID-19 pandemic as telehealth became increasingly integrated into routine care [33]. These regulatory and infrastructure shifts, along with clinical trial evidence, have advanced RPM from a sparsely used, research-focused tool to a potentially scalable model for routine clinical care. Nevertheless, while some costs may be offset through reimbursement or reductions in avoidable acute care use, financial sustainability will depend on local payer policies, patient eligibility, enrollment volume, and the extent to which remote monitoring can be seamlessly incorporated into existing clinical workflows.

### Future Directions

This pilot provides the foundation for a larger, adequately powered pragmatic trial that will evaluate the effectiveness, implementation, and economic implications of digitally supported adherence monitoring for patients receiving oral anticancer therapy. The next phase should test a standardized intervention that combines adherence assessment, tailored education, symptom monitoring, and risk-based clinical escalation, while comparing outcomes across health literacy, age, race and ethnicity, rurality, and digital access groups. Embedding the intervention in the EHR and conducting mixed methods evaluation with patients and clinical staff would help identify the workflow, staffing model, and alert thresholds needed for sustainable dissemination in both academic and community oncology settings.

### Limitations

A number of limitations should be acknowledged. First, this was an exploratory, post hoc analysis that was not prespecified in the trial protocol; therefore, the findings should be interpreted cautiously and viewed as hypothesis generating rather than confirmatory. Second, the study was limited to a small sample of English-speaking participants who had a mobile phone and were receiving care at a single NCI-designated comprehensive cancer center or at a large public hospital. As a result, the findings may not generalize to broader oncology populations, particularly patients who differ in socioeconomic background, digital access, digital literacy, language, or care setting. Given the relatively narrow population observed in this study, these findings should be interpreted cautiously when considering its generalizability among broader oncology populations. Third, although disease stage was similar across study arms, it was not included in the adjusted model because of the small sample size, and residual confounding by stage and treatment regimen remains possible. Fourth, the single-item health literacy screener is a simplified measure that only captures one dimension of health literacy and does not assess broader skills, such as numeracy, comprehension, or communication. Nevertheless, the measure has been validated across multiple clinical populations and correlates strongly with longer instruments, making it a practical screening tool for time-constrained clinical and research settings [20,21]. Finally, the small sample size combined with the strong skew toward the highest response category required us to collapse 4 response options into the lower health literacy group, which may have introduced misclassification and grouped participants with meaningfully different levels of skill [20]. As a result, the subgroup findings should be interpreted as hypothesis generating and based on a simplified health literacy classification rather than a comprehensive assessment. Future research should use larger, more diverse samples and more robust health literacy measures to characterize how health literacy relates to medication adherence and to assess whether remote monitoring is effective across different patient populations and care settings.

### Conclusions

In this pilot exploratory analysis, a remote monitoring intervention using a smart pill bottle and simple text-based symptom assessments was associated with higher predicted adherence among participants with lower health literacy but not among those with higher health literacy. Although the interaction test did not reach statistical significance, the magnitude and direction of the observed difference support further investigation of RPM as a promising approach to improve adherence among patients facing health literacy–related barriers. Given the small sample and post hoc design, findings should be interpreted cautiously. Future trials are needed to determine whether these effects are reproducible in more diverse patients with different socioeconomic backgrounds, digital literacy levels, and care settings and to clarify which intervention components are most beneficial. If confirmed, tailored remote monitoring could become a key component of a patient-centered oncology care aimed at reducing barriers to oral anticancer therapy adherence.

## Appendix

Multimedia Appendix 1 CONSORT-eHEALTH checklist (V 1.6.1).

## Abbreviations

CONSORT: Consolidated Standards of Reporting Trials
EHR: electronic health record
EUC: enhanced usual care
FPL: federal poverty level
HIPAA: Health Insurance Portability and Accountability Act
mHealth: mobile health
NCI: National Cancer Institute
RPM: remote patient monitoring

## Data Availability

The data generated or analyzed during this study are not publicly available due to privacy restrictions but are available from the corresponding author on reasonable request.
